# Metformin Enhances the Anti-Cancer Efficacy of Sorafenib via Suppressing MAPK/ERK/Stat3 Axis in Hepatocellular Carcinoma

**DOI:** 10.3390/ijms23158083

**Published:** 2022-07-22

**Authors:** Sumit Siddharth, Panjamurthy Kuppusamy, Qitong Wu, Arumugam Nagalingam, Neeraj K. Saxena, Dipali Sharma

**Affiliations:** 1 Department of Oncology, Johns Hopkins University School of Medicine, Baltimore, MD 21287, USA; ssiddha2@jhmi.edu (S.S.); qwu38@jhmi.edu (Q.W.); anagali1@jhmi.edu (A.N.); 2 Sidney Kimmel Comprehensive Cancer Center at Johns Hopkins, Baltimore, MD 21287, USA; 3 Department of Medicine, University of Maryland School of Medicine, Baltimore, MD 21201, USA; pkuppusamy@som.umaryland.edu; 4 National Cancer Institute, National Institutes of Health, Rockville, MD 20850, USA; neeraj.saxena@nih.gov

**Keywords:** hepatocellular carcinoma, combination treatment, sorafenib, metformin, MAPK, ERK, Stat3

## Abstract

Hepatocellular carcinoma (HCC) incidence, as well as related mortality, has been steadily increasing in the USA and across the globe, partly due to the lack of effective therapeutic options for advanced HCC. Though sorafenib is considered standard-of-care for advanced HCC, it only improves median survival by a few months when compared to placebo. Sorafenib is also associated with several unpleasant side effects that often lead to early abatement of therapy. Here, we investigate whether a combination regimen including low-dose sorafenib and a non-toxic dose of anti-diabetic drug metformin can achieve effective inhibition of HCC. Indeed, combining metformin with low-dose sorafenib inhibited growth, proliferation, migration, and invasion potential of HCC cells. We observed a 5.3- and 1.9-fold increase in sub-G1 population in the combination treatment compared to sorafenib alone. We found that the combination of metformin enhanced the efficacy of sorafenib and inhibited the MAPK/ERK/Stat3 axis. Our in vivo studies corroborated the in vitro findings, and mice harboring HepG2-derived tumors showed effective tumor reduction upon treatment with low-dose sorafenib and metformin combination. This work sheds light on a therapeutic strategy aiming to augment sorafenib efficacy or dose-de-escalation that may prove beneficial in circumventing sorafenib resistance as well as minimizing related side effects.

## 1. Introduction

The third leading cause of cancer-related deaths worldwide, primary liver cancer, ranks the sixth most commonly diagnosed cancer in 2022 [1,2]. Hepatocellular carcinoma (HCC) is the most common type of liver cancer, encompassing 75–85% of total cases [2], and its incidence and associated mortality have been steadily increasing in North America and some European countries [3]. Alcoholic liver disease, non-alcoholic fatty liver disease (NAFLD), metabolic syndrome, and viral hepatitis are among the commonly known risk factors for HCC [4,5,6]. Among these risk factors, alcoholic liver disease, that encompasses cirrhosis, alcoholic hepatitis, fibrosis, and steatosis [7] is responsible for 30% of HCC cases and HCC-related mortality [8]. NAFLD also shows a strong association with HCC. As the embodiment of metabolic syndrome, NAFLD significantly impacts the adult population in the USA [9,10], and patients with NAFLD demonstrate a higher incidence of HCC [11]. Viral hepatitis, caused by Hepatitis B virus or Hepatitis C virus, is another important risk factor. While individuals infected with Hepatitis B virus can develop HCC [12], an active Hepatitis C virus infection renders the patients 15–20-fold more likely to develop HCC [7]. Additional risk factors that favor the development of HCC include Hispanic ethnicity, obesity, male gender, and diabetes [13,14]. Notably, a retrospective cohort study [15] identified diabetes as a major risk factor that is directly related to the pathogenesis of HCC [16,17,18]. Further, meta-analyses reported that the increased HCC risk in patients with type 2 diabetes is unrelated to alcohol consumption or viral hepatitis [19,20]. Interestingly, long history of diabetes with increased exposure to sulphonyl urea and insulin treatment increases the risk of HCC [21]. Of note, metformin treatment is negatively correlated to HCC risk [22]. Due to poor surveillance and limited patient compliance, HCC is frequently diagnosed at advanced stages in patients with diabetes and chronic liver disease, and subsequently, upon diagnosis, effective treatment strategies are not available [8].

Therapeutic interventions for HCC vary by the stage of the disease. Hepatic resection or radiofrequency ablation (RFA) are among the major therapeutic strategies to combat the early stage of HCC [3]. Meanwhile, sorafenib is the mainstay drug for advanced HCC [23,24,25,26]. However, treatment outcomes are generally discouraging [18]. Among patients with advanced HCC, the median overall survival with sorafenib treatment is approximately 8 to 11 months [26]. Sorafenib is a tyrosine kinase inhibitor. By interfering with multiple kinases, sorafenib has the leverage to target several pathways, including the Ras/Raf/MAPK pathway, the vascular endothelial growth factor signaling pathway, and the platelet-derived growth factor signaling pathway [27]. However, it has been noted that sorafenib only imparts a few months increase in median survival in patients with advanced HCC compared to the placebo group [26,28]. Sorafenib is also associated with unpleasant side effects, such as acneiform rash, diarrhea, hypertension, and fatigue [29]. Combining sorafenib with surgery or transarterial chemoembolization does not yield substantial improvement in overall survival [30]. The benefits of sorafenib are restricted to 30% of advanced HCC patients who later develop drug resistance and adverse side effects [31].

An antidiabetic drug, metformin, has been explored for its anti-cancer ability [22,32,33,34]. Some studies have reported that metformin is associated with a decreased incidence of HCC among diabetic patients [35,36]. Metformin can interfere with the activation of ERK1/2 and JNK1/2 [37], which are important players in tumorigenesis [38] and proliferation [39]. In HCC, enrichment of uPA and MMP-9 is associated with poor prognosis [40,41], and their expression and secretion can be blocked by metformin [37]. Additionally, metformin’s leverage to lower glucose and sensitize insulin may slow the proliferation of premalignant hepatic lesions that flourish in an environment that is high in glucose or insulin. Importantly, many studies have reported metformin’s ability to inhibit tumor growth [42,43,44]. Metformin has been broadly recognized as a safe therapeutic [37], though a high dosage of metformin can also lead to lactic acidosis [45].

The present clinical scenario suggests the insufficiency of single-agent therapeutics for HCC and demands an urgent need for the combination treatment approach. As combination treatment targets multiple pathways either additively or synergistically, it strengthens the anti-cancer drug treatment regimen compared to monotherapy. Since effective an dose of sorafenib is linked with multiple side effects, sometimes leading to the discontinuation of treatment, we examined whether a combination regimen using half-doses of sorafenib and metformin would achieve effective HCC inhibition. Indeed, combining low doses of metformin and sorafenib proved effective in inhibiting key pathways linked with HCC growth and drug resistance as well as impeding tumor growth in mice. 

## 2. Results

### 2.1. Combination Treatment with Metformin and Morafenib Showed Superior Effects in HCC Cells

**Figure 1:**
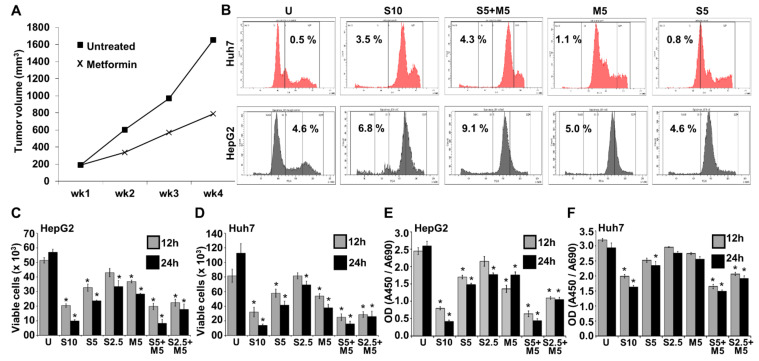
Metformin improved the efficacy of sorafenib in HCC cells. (**A**) Line graph represents the tumor progression curve of HepG2 -derived tumors treated with vehicle and metformin (200 mM). (**B**) Cell cycle analysis of HepG2 and Huh7 cells treated with 10 µM sorafenib (S10), 5 µM sorafenib (S5), 5 mM metformin (M5), and the combination of 5 µM sorafenib and 5 mM metformin (S5 + M5). (**C**–**F**) HepG2 and Huh7 were treated with 10 µM, 5 µM, and 2.5 µM sorafenib (S10, S5, S2.5) and/or 5 mM metformin (M5) either alone or in combination for 12 and 24 h, respectively. (**C**,**D**) Trypan blue dye exclusion assay was performed to assess cell survival. Graphs represent the number of viable cells. (**E**,**F**) XTT assay was performed to assess cell viability. Graphs represent the optical density of absorbance at 450/690 of the samples. * *p* < 0.05.

Metformin, a common anti-diabetic drug, has shown anti-cancer effects in many types of cancer, hence, we first examined the effectiveness of metformin on HCC tumor growth. Metformin treatment inhibited the growth of HepG2-derived tumors compared to vehicle-treated group (Figure 1A). Since sorafenib treatment is associated with therapy-limiting severe side effects, we aimed to explore whether a combination regimen of low-dose sorafenib and metformin is as effective as the high-dose sorafenib alone. Cell cycle analysis of HCC cells treated with high/low-dose sorafenib, metformin, and the combination of low-dose sorafenib and metformin exhibited increased sub-G1 population with low-dose sorafenib alone treatment, which is further enhanced with a low-dose sorafenib-metformin combination, compared to vehicle-treated cells (Figure 1B). A 5.3- and 1.9-fold increase in sub-G1 population was noted in the combination treatment group (sorafenib 5 μM + metformin 5 mM) compared to 5 μM sorafenib monotherapy group in HepG2 and Huh7 cells, respectively, and is equivalent to high-dose sorafenib group (Figure 1B). Sorafenib treatment also led to an S phase arrest in liver cancer cells (Figure 1B). The combination treatment with low-dose sorafenib and metformin significantly reduced the number of viable HepG2 and Huh7 cells (Figure 1C,D) and was comparable to high-dose sorafenib alone. A dose-dependent and statistically significant reduction in cell growth was noted in HCC cells treated with low-dose sorafenib and metformin combination compared to either monotherapy (Figure 1E,F).

### 2.2. Decreased Invasion and Migration Potential Was Observed with Metformin and Sorafenib Combination

**Figure 2:**
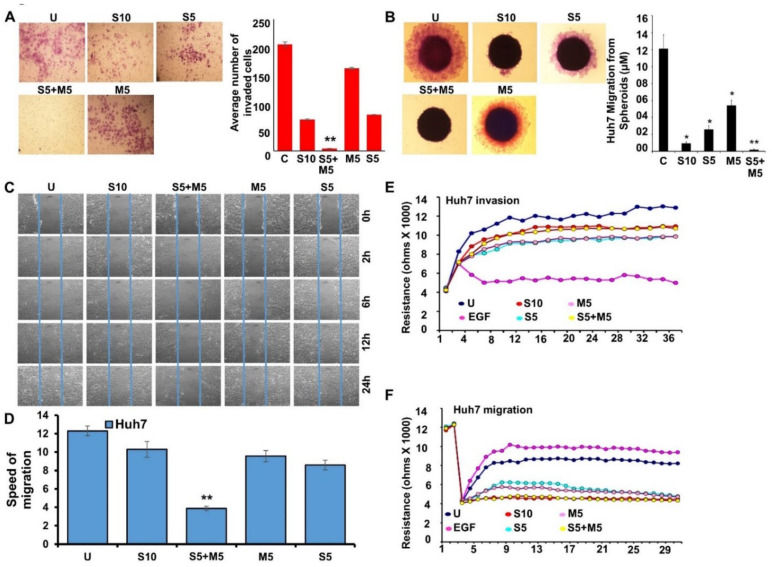
Combination treatment with metformin and sorafenib decreased the invasive and migratory potential of HCC cells. (**A**–**F**) Huh7 cells were treated with 10 µM sorafenib (S10), 5 µM sorafenib (S5), 5 mM metformin (M5), and the combination of 5 µM sorafenib and 5 mM metformin (S5 + M5) and subjected to various assays. (**A**) Representative images of Huh7 cells invaded through matrigel. Graph shows the number of Huh7 cells invaded through matrigel chambers. (**B**) Representative images of Huh7 spheroids incubating in treatments as indicated. Graph showing the distance migrated from the spheroids. (**C**,**D**) Representative images of Huh7 cells migrating through a scratch in a wound-healing assay. Images were captured at 0, 2, 6, 12, and 24 h. Graph showing the speed of migration of Huh7 cells treated with different drug combinations as indicated. (**E**) HUVEC monolayer was challenged with Huh7 cells in the presence of drugs as indicated. Change in resistance is shown here. (**F**) Huh7 cells were cultured on fibronectin-coated ECIS cellware and treated with drugs as indicated. Change in resistance is shown here as cells migrate on the electrode. * *p* < 0.05; ** *p* < 0.001.

Further, we questioned whether combining metformin with low-dose sorafenib impacted the invasive and migratory potential of liver cancer cells. Metformin and low-dose sorafenib alone reduced the invasion of Huh7 cells in comparison to untreated controls. More effective inhibition of invasion was achieved by combining metformin with low-dose sorafenib compared to high-dose sorafenib (Figure 2A). Next, to examine the modulation of the migratory potential of Huh7 cells in response to sorafenib and metformin, we performed spheroid-migration and scratch-migration assays. As single treatments, low-dose sorafenib and metformin inhibited migration, but a greater degree of inhibition was observed by combining low-dose sorafenib and metformin, and it was equivalent to high-dose sorafenib (Figure 2B). Similar findings were observed with scratch-migration assay, where a combined treatment with low-dose sorafenib and metformin was more effective than high-dose sorafenib as well as single treatments with low-dose sorafenib and metformin (Figure 2C,D). Further, we performed a quantitative real-time impedance assay using electric cell-substrate impedance sensing (ECIS) in order to evaluate the invasion and migration of Huh7 cells over the ECIS plates in response to sorafenib and metformin treatments. In the ECIS-invasion assay, EGF-treated group showed a lower resistance as more Huh7 cells invade through the monolayer of HUVEC cells on the electrode, shifting the HUVEC layer. Increased resistance was observed in metformin and sorafenib treatment groups compared to the EGF-treated group as fewer Huh7 cells invaded through the HUVEC cell monolayer (Figure 2E). In the ECIS-migration assay, Huh7 cells treated with low-dose sorafenib and metformin showed a decrease in resistance, showing decreased migration in comparison to untreated cells (Figure 2F).

### 2.3. Metformin and Sorafenib Combination Inhibited Key Signaling Molecules

**Figure 3:**
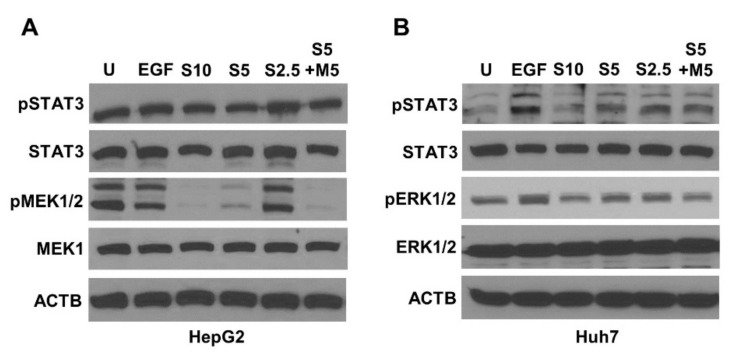
Combination treatment with metformin and low-dose sorafenib decreased the phosphorylation of signaling molecules. (**A**,**B**) HepG2 and Huh7 cells were treated with 10 µM sorafenib (S10), 5 µM sorafenib (S5), 2.5 µM sorafenib (S2.5), and the combination of 5 µM sorafenib and 5 mM metformin (S5 + M5) and subjected to Western blot analysis. Images of immunoblots of phospho-STAT3, STAT3, phospho-MEK1/2, and MEK1/2 in HepG2 cells, and phospho-STAT3, STAT3, phospho-ERK1/2, and ERK1/2 in Huh7 cells are shown. ACTB serves as an internal control.

Next, we investigated the levels of phosphorylated STAT3, MEK1/2, and ERK1/2 upon sorafenib and metformin treatment alone as well as in combination. Both sorafenib and metformin suppressed the activation of MEK1/2, ERK1/2, and Stat3, with a stronger inhibition observed in response to combination treatment (S5 + M5). We found an effective inhibition of phospho-MEK1/2 in S5 + M5 group comparable to S10 group in HepG2 cells (Figure 3A). A similar reduction was also observed in the expression of phospho-ERK1/2 in Huh7 cells (Figure 3B). Taken together, our findings suggest a potential signaling interplay where metformin is inducing the anti-cancer efficacy of sorafenib.

### 2.4. Metformin and Low-Dose Sorafenib Combination Inhibited HCC Tumor Growth In Vivo

**Figure 4:**
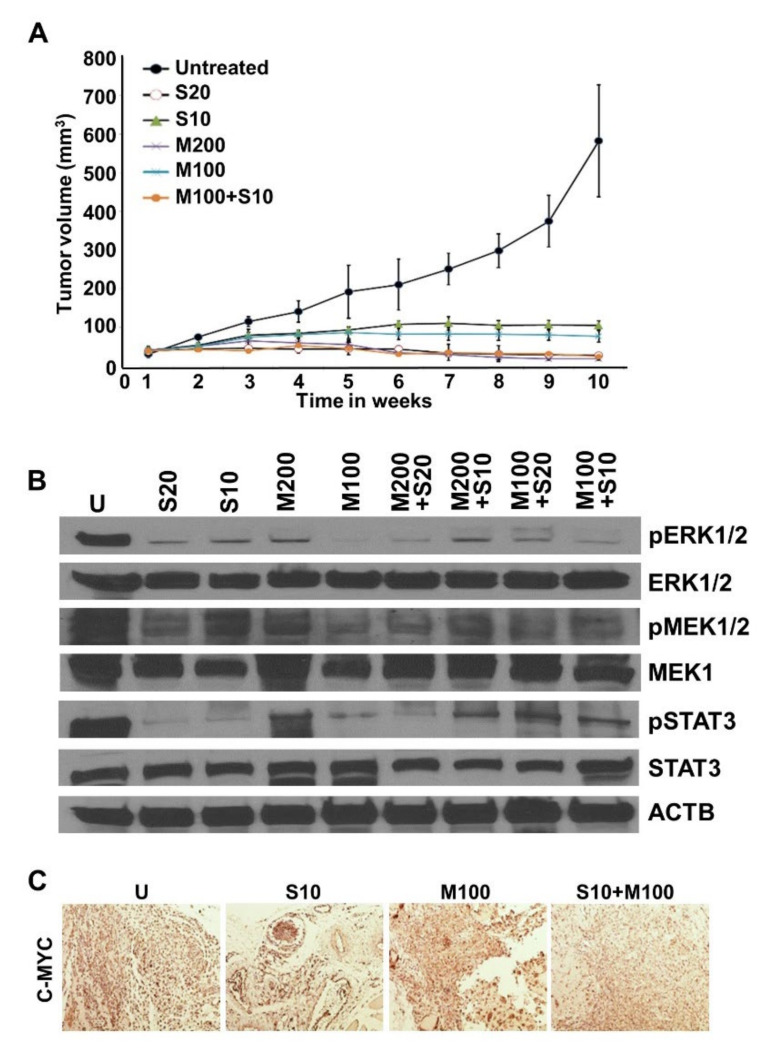
Combination regimen of metformin and low-dose sorafenib inhibited HCC tumor growth. (**A**) Line graph represents tumor progression curve of HepG2 tumors treated with sorafenib (S) (20 µM and 10 µM), metformin (M) (200 mM and 100 mM), and the combination of sorafenib + metformin (M100 + S10). (**B**) Immunoblot analysis of vehicle and drug-treated HepG2 derived tumor extracts for the detection of phospho-MEK1/2, MEK1/2, phospho-ERK1/2, ERK1/2 and phospho-STAT3, STAT3. ACTB served as the internal control. (**C**) Immunohistochemical analysis of C-MYC in the paraffin-embedded tissue sections of vehicle- and drug-treated HepG2 derived tumors.

Our in vitro findings encouraged us to investigate the significance of metformin and low-dose sorafenib combination in vivo. Mice bearing HepG2-derived tumors exhibited decreased tumor growth in drug-treatment groups in comparison to the vehicle-treatment group. Low-dose sorafenib and metformin combination group showed efficient tumor inhibition comparable to high-dose sorafenib. Importantly, the extent of tumor volume reduction attained with S20 and M200 treatment level was attained at the half dose (S10 + M100) in the combination group (Figure 4A). Immunoblot analyses of tumor xenografts corroborated our in vitro findings and revealed decreased expression of phosphorylated ERK1/2, MEK1/2, and Stat3 in the combination treatment group compared to either monotherapy (Figure 4B). Immunohistochemical analyses of the tumor samples showed reduced expression of C-MYC in the combination treatment groups compared to vehicle- or monotherapy-treated groups (Figure 4C). Collectively, these data show that a combination regimen of low-dose sorafenib and metformin is as effective as high-dose sorafenib (standard dose) in achieving effective inhibition of growth, invasion, and migration of HCC cells as well as blocking HCC tumor progression.

## 3. Discussion

The establishment of sorafenib as a standard drug for advanced HCC is considered a turning point despite only a few months increase in median survival of patients [23,24,25,26]. Multiple side effects are also associated with sorafenib that may lead to early abatement of therapy [18]. In addition, the development of sorafenib resistance poses another clinically relevant problem. Therapeutic strategies aiming to augment sorafenib efficacy or dose-de-escalation may prove beneficial in circumventing resistance as well as reducing side effects. Over the years, multiple strategies have been examined, combining various existing or new drugs with sorafenib. Combining Ivermectin, an FDA-approved anti-parasitic drug, with sorafenib led to a more effective reduction of HCC tumors and inhibition of mTOR/STAT3 pathway along with reduced stemness [46]. Targeting YAP/TAZ pathway with a YAP1 inhibitor CA3 in combination with sorafenib showed inhibition of HCC cells and tumor spheroids [47]. PI3K/mTOR presents an important oncogenic pathway in HCC whose inhibition has shown anti-cancer potential. PI3K/mTOR inhibitor GSK1059615 and sorafenib-loaded PLGA-PEG-mal deblock copolymer proved efficacious in activating NF-kB, inhibiting programmed cell death ligand 1 (PD-L1), and ameliorating sorafenib resistance [48]. In addition to targeted inhibition of signaling pathways to increase sorafenib efficacy, combining a natural product, such as ginsenoside Rg3, with sorafenib also showed enhanced inhibition of HCC cells [49]. Several efforts are underway to improve HCC therapy using immune checkpoint inhibitors as single agents and in combination. Phase I/II CheckMate 040 and the KEYNOTE-224 phase II trials queried the efficacy of second-line nivolumab and pembrolizumab. CheckMate 459 and the KEYNOTE-240, comparing nivolumab to sorafenib and pembrolizumab to best supportive care, showed no major advances. However, IMbrave 150 trial examining the efficacy of atezolizumab and bevacizumab in comparison to sorafenib showed improved overall survival [50,51]. Of interest, HCC patients treated with metronomic capcitabine exhibit potential anti-HCC effects, but further studies are required [52]. A comparison of sorafenib with a combination of cabozantinib plus atezolizumab in advanced HCC showed longer primary progression-free survival while additional studies are ongoing [53]. Multiple studies are exploring a number of combination regimens with the goal of improving the prognosis for HCC patients with advanced disease.

In the present study, we evaluated the preclinical efficacy of low-dose sorafenib and metformin combination in vitro and in vivo and found that this combination regimen decreases the growth, proliferation, invasion, and migration potential as effectively as the high-dose sorafenib. The extent of tumor inhibition observed with high-dose sorafenib and high-dose metformin as single treatments was achieved by combining half doses of sorafenib and metformin. Recently, Ling and colleagues evaluated the efficacy of the combination treatment of metformin and sorafenib in HCC and concluded that the combination treatment induces apoptosis and inhibits cell proliferation both in vitro and in vivo [54]. The synergistic effect of metformin and sorafenib seemed to induce AMPK signaling in HCC cells [55,56]. Harati and colleagues examined the effect of a clinically relevant dose of metformin (50 mg/kg/d) on the antitumoral effects of sorafenib (15 mg/kg/d) using different modes of administration (concomitant versus sequential). They observed significantly reduced tumor volumes when metformin was administered concomitantly in comparison to the sorafenib-alone group with no antitumoral effects in the sequential group [18]. A recent study showed that although sorafenib treatment reduced tumor volume and prolonged median survival but increased lung metastasis. However, the combination treatment of sorafenib and metformin reduced tumor volume as well as lung metastasis, and prolonged median survival [57]. Combination treatment with sorafenib and metformin inhibited cell proliferation and invasion potential of MHCC97H cells [57]. However, a clinical study of 93 patients where 31 patients administered metformin simultaneously as they were suffering from diabetes mellitus, revealed a 2.6 months median progression-free survival compared to 5 months median progression-free survival in the sorafenib-alone group. At the same time, 15.1 months median progression-free survival was observed in patients administered with sorafenib alone compared to 10.4 months of median free survival in the combination group [58]. In the patient population with hepatic resection and liver transplantation, metformin did not show synergistic effects with sorafenib [59]. Although combination strategies using a full dose of two drugs or more show increased efficacy, they often fail owing to increased side effects. Our study evaluated a feasible dose de-escalation strategy involving two FDA-approved drugs, sorafenib and metformin, which can potentially reduce the side effects associated with these drugs as well as provide increased efficacy. Our results are supported by a recent study showing that reduced doses of sorafenib along with standard doses of atorvastatin and metformin led to reduced sorafenib-related side effects in advanced HCC [60].

The contribution of MAPK/ERK signaling in tumorigenesis is undeniable, but its role in HCC is less explored. Irrespective of the low frequency of RAS and RAF mutations in HCC, activated MAPK/ERK signaling is commonly seen in HCC patients [61]. Active MAPK/ERK signaling is observed in almost 50% of early-stage HCC patients and ~100% of advanced-stage HCC patients [62]. Epidermal growth factor (EGF) and transforming growth factor alpha (TGF-alpha) bind to EGFR and trigger MAPK/ERK pathway, thus contributing to MAPK/ERK activation [63]. Multiple lines of evidence suggest induced expression of EGF and TGF-alpha in early-stage hepatocarcinogenesis, demonstrating the possible role of MAPK/ERK pathway in tumor progression of hepatocytes [64,65]. Sorafenib inhibits PI3K/AKT, MAPK signaling cascades leading to mTOR inhibition [58,66]. Sorafenib also induces AMPK via LKB1, eventually blocking mTOR signaling [58,66,67]. A recent study demonstrated that the anti-proliferative and anti-angiogenic effects of metformin are via inhibition of mTOR pathway in an AMPK-dependent, as well as independent manner [67]. These findings suggest that both sorafenib and metformin converge onto the same downstream pathway. Our findings indicate that while both metformin and sorafenib inhibited MEK/ERK/Stat signaling, combination treatment had a higher inhibitory effect at a lower dose than either monotherapy. While our study shows the benefit of combining low doses of metformin and sorafenib to achieve effective HCC inhibition, additional pharmacokinetics and pharmacodynamics studies as well as animal experiments are needed to support the clinical development of this regimen. If successful, combining low doses of metformin and sorafenib may improve the outcomes for patients with advanced HCC.

## 4. Materials and Methods

### 4.1. Cell Culture and Reagents

Human hepatocellular carcinoma cells (HepG2 and Huh7) were maintained and propagated following the published protocol [68]. Briefly, the cells were grown in DMEM containing 10% FBS and 1% antibiotic/antimycotic and maintained in a humidified atmosphere of 5% CO_2_ at 37 °C. Sorafenib (S7397) and Metformin (S5958) were procured from Selleck Chemicals (Selleck Chemicals, Houston, TX, USA). Anti-pSTAT3, anti-STAT3, anti-pMEK1/2, anti-MEK1/2, anti-pERK1/2, anti-ERK1/2, and anti-CMYC were procured from Cell Signaling Technology (Cell Signaling Technology, Beverly, MA, USA). 2,3-bis(2-methoxy-4-nitro-5-sulfophenyl)-2H-tetrazolium-5-carboxyanilide (XTT) was purchased from Roche Applied Science (Roche Applied Science, Indianapolis, IN, USA). For electric cell-substrate impedance sensing (ECIS) migration assay, ECIS cell cultureware was purchased from Applied Biophysics (Applied Biophysics, Troy, NY, USA).

### 4.2. XTT Cell Viability Assay

The viability of HCC cells upon treatment with sorafenib, metformin and sorafenib + metformin was determined using XTT (2,3-bis(2-methoxy-4-nitro-5-sulfophenyl)-2H-tetrazolium-5-carboxyanilide) cell viability assay following the manufacturer’s instructions. Briefly, 5 × 10^3^ cells per well were seeded in a 96-well plate for 24 h and treated with the indicated concentrations of sorafenib, metformin, and sorafenib + metformin for 48 h. After the incubation period, XTT reagent was supplemented to attain the final concentration of 0.3 mg/mL and incubated for 4 h at 37 °C. Then, the absorbance was measured at 450 nm and 690 nm using microplate reader (SPECTRAmax PLUS, Molecular Devices, Sunnyvale, CA, USA).

### 4.3. Trypan Blue Dye Exclusion Assay

Trypan blue dye exclusion assay was performed to assess the number of viable cells upon sorafenib, metformin treatment either alone or in combination. Drug-treated cells were trypsinized, resuspended in trypan blue dye, counted, and viable cells were presented graphically [69].

### 4.4. HepG2 Xenografts in Nude Mice

HepG2 xenografts were established following our previously published protocol [70]. HepG2 cells (5 × 10^6^ cells) were resuspended in 100 µL 1:1 ratio mixture of matrigel and 1X PBS. It was then injected subcutaneously into the right gluteal region of 4–6-week-old, male, athymic nude mice for tumor development. After two weeks, the tumor-bearing mice were randomized into vehicle (PBS) and metformin-treated groups. In another set of experiments, the tumor bearing mice were randomized into vehicle and 8 treatment groups with various combinations of metformin (M) and sorafenib (S) as S20, S10, M200, M100, S20 + M100, S20 + M200, S10 + M100, and S10 + M200. Tumor volumes were measured twice a week, using caliper and plotted graphically. After 5 weeks of treatment, the mice were sacrificed, tumors were dissected and subjected to further processing for immunoblotting and immunohistochemical staining. All animal studies were approved by the Institutional Animal Care and Use Committee, University of Maryland School of Medicine, Office of Welfare Assurance, Baltimore, Maryland.

### 4.5. Cell Cycle Analysis

To determine the regulation of cell cycle in sorafenib-, metformin- and sorafenib + metformin-treated cells, cell cycle analysis was performed following the previously published protocol [71]. In brief, cells were exposed to different drugs for 48 h. After the incubation period, the cells were trypsinized, washed with PBS containing 0.05% RNAse-A, fixed with 70% ethanol, and stained with 50 µg/mL propidium iodide. The stained cells were acquired by flow cytometry with an event count of 10,000 cells per sample. The DNA content was analyzed using FACSDiva software.

### 4.6. Immunoblotting

The whole cell lysates were prepared using a modified RIPA lysis buffer and quantified using the Bradford protein assay kit. Equal amounts of protein lysates were resolved on SDS-PAGE, transferred onto PVDF membrane, and blotted with respective antibodies.

### 4.7. Spheroid Migration Assay

A spheroid migration assay was performed following the published protocol [72]. In brief, 2 × 10^4^ cells were grown as spheroids on agarose-coated plates using an orbital shaker at 500 rpm. Intact spheroids were transferred to 12-well plates and treated as indicated for 48 h. After the incubation period, spheroids were fixed with 10% buffered formalin in PBS and stained with crystal violet. The migration of cells from spheroids was observed under a light microscope. Data were then quantified and plotted graphically. 

### 4.8. Matrigel Invasion Assay

The invasive potential of untreated and treated HCC cells was analyzed by matrigel invasion assay following our previously published protocol [73]. 2 × 10^4^ cells were seeded in the matrigel invasion chambers in serum-free media while the lower chambers were supplemented with serum-containing DMEM. Cells invaded through matrigel were fixed, stained with crystal violet (0.1% in 20% methanol), and analyzed under the microscope. Microscopic images were quantified and data were presented graphically.

### 4.9. Electric Cell-Substrate Impedance Sensing (ECIS)-Migration and Invasion Assay

ECIS-invasion and ECIS-migration assays were performed to measure the migration and invasion potential of HCC cells in real time upon indicated treatment following our previously published protocol [74]. ECIS technology (Applied BioPhysics, Troy, NY, USA) was used for the wound-healing or migration assay. HepG2 and Huh7 cells were grown to confluence on the ECIS plates, and submitted to an elevated voltage pulse of 40 kHz frequency, 3.5-V amplitude, and 30-s duration to create a wound over the small active electrode. The plates were treated with sorafenib and metformin as indicated and the wound healing was monitored by continuous resistance measurements. *For invasion assay*, ECIS plates were seeded with 1 × 10^5^ human umbilical vein endothelial cells (HUVEC) and allowed to spread over the electrode. HUVEC layer was challenged with HepG2 or Huh7 cells followed by sorafenib and metformin treatment as indicated. The impedance of the challenged endothelial cell layer was monitored via ECIS.

### 4.10. Scratch Migration Assay

The monolayer migration of untreated and treated HCC cells was analyzed using a Scratch migration assay following our previously published protocol [73]. Trypsinized cells were seeded in 12-well plates to attain 90% confluence. A 1-mm wide scratch was made across the cell monolayer using a sterile pipette tip (0.5 to 10 µL). Each well was washed twice with PBS, and treated as indicated. Plates were photographed immediately for 0 h followed by 2, 6, 12- and 24-h intervals at the identical location of the initial image. Wound closure was quantified using ImageJ software and plotted using MS Excel.

### 4.11. Immunohistochemical Staining

Paraffin-embedded tumor tissues were rehydrated and probed with anti-C-MYC antibody followed by HRP-conjugated secondary antibody and developed using DAB-peroxidase substrate kit (SK-4100, Vector laboratories, Newark, CA, USA). Images were captured using a Nikon microscope at 20× magnification.

### 4.12. Statistical Analyses

All experiments were performed thrice in triplicates and statistical analysis was performed using Microsoft Excel software. Student *t* test and two-tailed distribution were used to analyze significant differences and the results were considered to be statistically significant if *p* < 0.05. Data were expressed as mean ± se between triplicate experiments performed thrice. For the animal studies, mean tumor volume between different groups was compared with analysis of variance with repeated measurements. The overall *p*-value for testing for differences between at least two groups is *p* < 0.001.

## 5. Conclusions

While the mortality associated with all other cancers is on a decline owing to better risk-prediction modeling, insightful prevention strategies, early detection, and effective treatment regimens, HCC-associated mortality has been steadily increasing in the USA and all over the globe. Treatment options include sorafenib for advanced HCC but is marred by limited efficacy and many side effects. We present that combining low doses of sorafenib and metformin yields more effective HCC inhibition in mice and inhibits key signaling molecules. Future clinical studies may explore the efficacy of half-doses of metformin and sorafenib in patients with advanced HCC and show the efficacy of this low-dose combination regimen.

## Data Availability

Not applicable.
